# Environmental Acidification Drives *S. pyogenes* Pilus Expression and Microcolony Formation on Epithelial Cells in a FCT-Dependent Manner

**DOI:** 10.1371/journal.pone.0013864

**Published:** 2010-11-05

**Authors:** Andrea G. O. Manetti, Thomas Köller, Marco Becherelli, Scilla Buccato, Bernd Kreikemeyer, Andreas Podbielski, Guido Grandi, Immaculada Margarit

**Affiliations:** 1 Novartis Vaccines and Diagnostics, Siena, Italy; 2 Institute of Medical Microbiology, Virology and Hygiene, Rostock, Germany; National Institutes of Health, United States of America

## Abstract

Group A Streptococcus (GAS, *Streptococcus pyogenes*) is a Gram-positive human pathogen responsible for a diverse variety of diseases, including pharyngitis, skin infections, invasive necrotizing fasciitis and autoimmune sequelae. We have recently shown that GAS cell adhesion and biofilm formation is associated with the presence of pili on the surface of these bacteria. GAS pilus proteins are encoded in the FCT (Fibronectin- Collagen-T antigen) genomic region, of which nine different variants have been identified so far. In the present study we undertook a global analysis of GAS isolates representing the majority of FCT-variants to investigate the effect of environmental growth conditions on their capacity to form multicellular communities. For FCT-types 2, 3, 5 and 6 and a subset of FCT-4 strains, we observed that acidification resulting from fermentative sugar metabolism leads to an increased ability of the bacteria to form biofilm on abiotic surfaces and microcolonies on epithelial cells. The higher biofilm forming capacity at low environmental pH was directly associated with an enhanced expression of the genes encoding the pilus components and of their transcription regulators. The data indicate that environmental pH affects the expression of most pilus types and thereby the formation of multicellular cell-adhering communities that assist the initial steps of GAS infection.

## Introduction

*Streptococcus pyogenes* (Group A *Streptococcus*, GAS) is a Gram-positive pathogen that colonizes and invades human epithelia causing an array of diseases with very different symptoms, ranging from mild pharyngitis and impetigo to severe necrotizing fasciitis and autoimmune sequelae. The ability of this pathogen to colonize and persist in distinct host sites, like the throat and the skin, and to trigger infections with very diverse clinical manifestations, relies on a wide range of virulence factors produced by the different isolates [1], [2], and on complex regulatory networks which modulate gene expression in response to fluctuating environmental conditions. Indeed, parameters like temperature, pH, O_2_, metal ion concentration, carbohydrate availability, salt, reactive oxygen species and antibacterial molecules have vast effects on GAS protein expression [3], [4], [5], [6], [7].

To initiate colonization of the human host, GAS adheres to epithelial cell surfaces, where it can form biofilm-like microbial communities which enhance resistance to host defenses and to nutrient deprivation [8], [9], [10], [11]. Several bacterial surface-associated molecules have been shown to be involved in these processes, among which are the fibronectin- binding adhesins F1 and F2 [12], [13] and the components of pilus-like structures [14], [15], all encoded in the genomic FCT region [16]. According to divergent gene organization and sequences, nine FCT variants have been identified [17], [18], which have been associated to strain tropism for specific infection sites [17].

To gain insight into the basis of FCT-specific tissue preferences, we have recently investigated the relationship between FCT-type and biofilm formation on abiotic surfaces using a collection of clinical GAS isolates [19]. Thereby, it was observed that FCT-1 strains formed biofilms independently of the culture medium used for their growth, whereas the biofilm forming capacity of strains belonging to other FCT variants showed a clear dependence on the growth medium.

Based on the differences in chemical composition of the media used in that study, here we analyzed a subset of isolates to investigate whether the presence of sugar in the growth medium, could play a role in the capacity of the different FCT-types to form microbial communities. We observed that a pH decrease associated with sugar metabolism led bacteria belonging to FCT-types 2, 3, 5, and 6 and a subgroup of FCT-4 strains, to grow in biofilm-like microbial communities. Conversely, FCT-1 strains formed biofilms independently of the presence of sugar or environmental pH. This pH-dependent phenotype variation appeared to be associated with a differential expression of some of the FCT-encoded genes in response to pH. The biofilm forming capacity was rendered pH-independent in recombinant FCT-3 M3 strains expressing FCT-3 pili under a constitutive promoter, indicating a direct link between pH-dependent pilus expression and biofilm formation by Group A *Streptococci*.

## Results

### Glucose affects biofilm formation by *S. pyogenes* in a FCT-dependent manner

**Figure 1:**
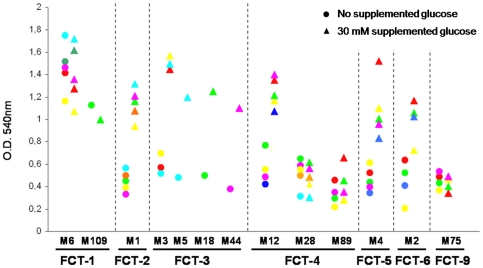
Effect of glucose on the biofilm forming capacity of GAS isolates belonging to different FCT-types. Forty-four GAS clinical isolates belonging to 7 FCT-types were grown under static conditions on 24-well polystyrene plates for 12 hours in non-buffered C medium without supplemented glucose (circles) or in the same medium added with 30 mM glucose (triangles); crystal violet stained, surface-attached bacteria were quantified by solubilizing the dye and measuring the absorbance at 540 nm; each dot represents the mean value of three independent experiments performed for each of the 44 isolates.

To investigate the influence of glucose on the capacity of certain GAS strains to form multicellular communities on abiotic surfaces, we performed classical biofilm plate assays using a selection of 44 isolates belonging to 13 different M types and to 7 FCT variants [19]. Bacteria were grown under static condition, either in a medium with very low sugar concentration (C medium, see Materials and Methods) or in the same medium supplemented with 30 mM glucose, and surface-attached bacteria were quantified after 12 hours. As shown in Figure 1, FCT-1 strains (serotypes M6 and M109) formed biofilm irrespective of the presence of glucose, whereas strains belonging to FCT-2 (M1), FCT-3 (M3, M5, M18, M44), FCT-5 (M4) and FCT-6 (M2) variants, plus a subset of FCT-4 strains belonging to the M12 serotype, showed increased biofilm formation when 30 mM glucose was added to the culture medium. Finally, a subgroup of M serotypes belonging to FCT-4 (M28 and M89) and to FCT-9 (M75) did not form biofilm at any of the investigated growth conditions. The incapacity of M28, M89 and M75 strains to form biofilm in C medium at any glucose concentration was consistent with our previous study showing the low capacity of those isolates to form biofilm in rich media [19].

Further experiments using C medium with increasing glucose concentrations from 10 to 70 mM, indicated that, in all sugar-dependent biofilm formers, maximum crystal violet staining intensity was reached in wells where bacteria were grown at 30 mM or higher glucose concentrations (data not shown). In control experiments, no difference in the cell division rate of any of the GAS strains was observed during planktonic growth in medium without added sugar or medium supplemented with 30 mM glucose (data not shown).

In conclusion, all tested GAS isolates belonging to FCT-types 2, 3, 4, 5, 6 capable of forming biofilm expressed this phenotype in a glucose-dependent manner.

### pH affects biofilm formation by *S. pyogenes* in a FCT-dependent manner

**Figure 2:**
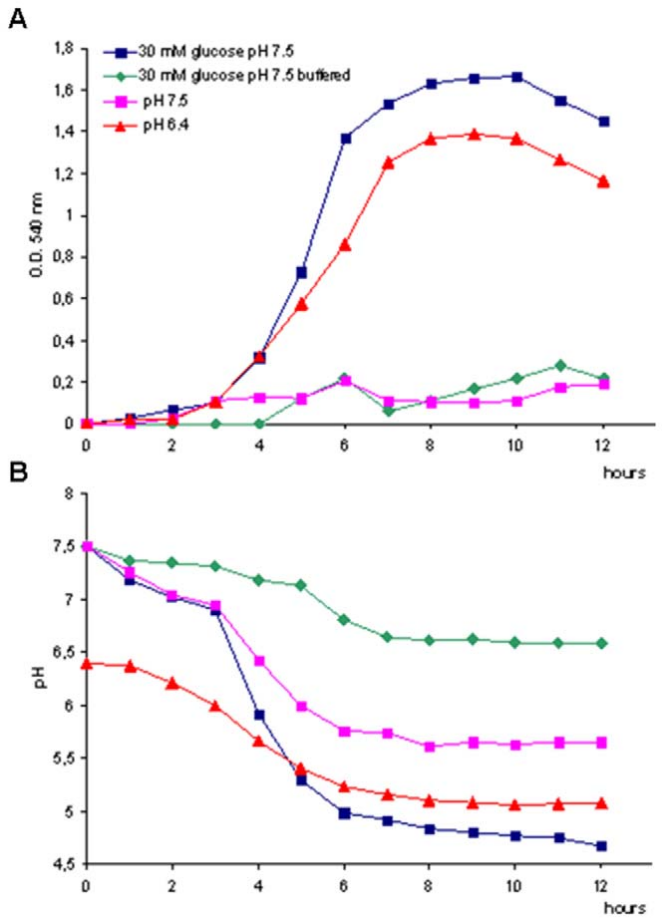
Effect of pH on the biofilm forming capacity of GAS FCT-3 isolate 43_M3. **A**) Time-course biofilm plate assay using GAS strain 43_M3 (FCT-3) under four different growth conditions: non-buffered C medium pH 7.5; non-buffered C medium pH 7.5 supplemented with 30 mM glucose; buffered C medium pH 7.5 supplemented with 30 mM glucose; non-buffered C medium pH 6.4. **B**) pH variation in parallel experiments using 43_M3 (FCT-3) under the same growth conditions (colors matching those of panel A).

The FCT-related behavior described above was also observed when fructose or mannose was used instead of glucose (data not shown). It is well known that during *S. pyogenes* growth fermentative metabolism results in the accumulation of organic acids generated as end products, leading to auto acidification [20]. Therefore, we reasoned that the pH decrease associated with sugar conversion to organic acids could be the direct cause of the observed effect on biofilm formation. To investigate this possibility, we carried out time-course biofilm assays with the glucose-dependent biofilm former FCT-3 strain 43_M3, using four different types of media. In particular, in addition to the two previously tested conditions (i.e. non-buffered media at pH 7.5 without added glucose or supplemented with 30 mM glucose), bacteria were incubated in phosphate-buffered medium at pH 7.5 supplemented with 30 mM glucose and in non-buffered medium at pH 6.4 without added sugar. For each growth medium, we measured both biofilm formation (Figure 2A) and the pH shift (Figure 2B) at different time points over a time span of 12 hours.

After 5 hours of incubation, we observed a strong biofilm increase when bacteria were grown in non-buffered medium at pH 7.5 in the presence of 30 mM glucose, but not in the same medium at pH 7.5 without sugar supplement. Remarkably, biofilm was formed when bacteria were grown at a starting pH of 6.4, even without supplemented glucose. Conversely, the biofilm-boosting effect of glucose was not apparent when the medium was buffered, allowing to rule out a catabolite repression effect for the observed biofilm phenotype.

In parallel assays, we measured the pH variation in each of the four growth conditions. As shown in Figure 2B, the presence of sugar in non-buffered medium at pH 7.5 resulted in a drop of the culture pH to about 5.0 after 6 hours, which matched an abrupt biofilm increase up to optical density at 540 nm (OD_540_) of 1.6. Likewise, bacterial growth in unbuffered medium without supplemented glucose at a starting pH of 6.4 lowered the pH of the medium to about 5.2 after 6 hours, leading to the formation of a quantifiable biofilm up to an OD of 1.4. On the contrary, bacteria grown at pH 7.5, both without sugar or using glucose-supplemented phosphate-buffered medium, were not able to reduce the pH to values under 5.7, and did not form biofilm at all. Control experiments showed that the four different investigated media allowed comparable bacterial growth in suspension (data not shown). Similar results as for 43_M3 (FCT-3) were obtained with FCT-2 strain 51_M1 grown in the same four types of media, while the FCT-1 27_M6 strain formed biofilm under all tested conditions (data not shown).

**Figure 3:**
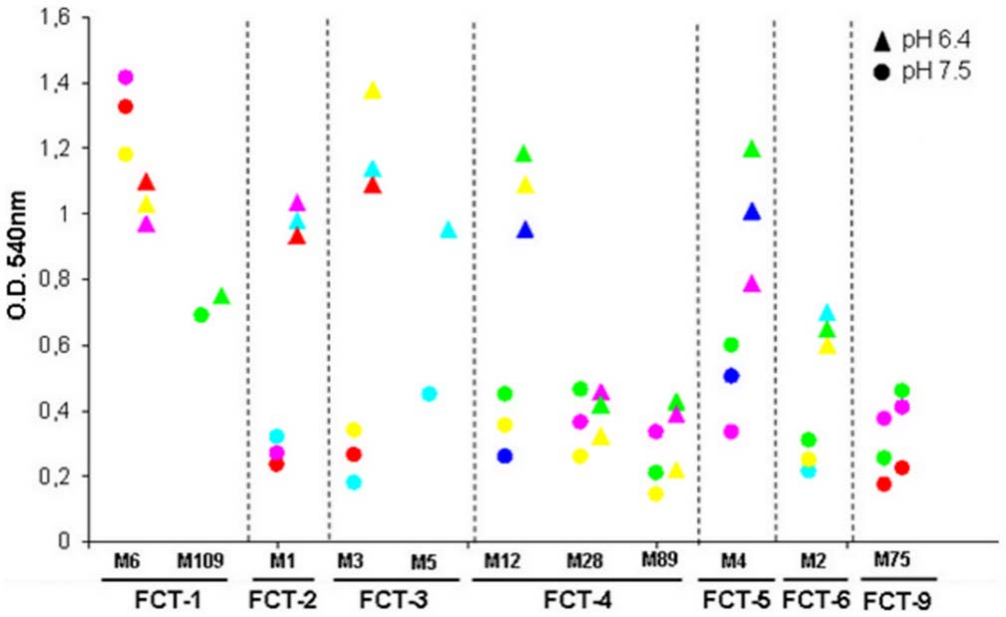
Effect of pH on the biofilm forming capacity of GAS isolates belonging to different FCT-types. Determination of biofilm formation by 29 GAS clinical isolates grown for 12 hours in 24-well polystyrene plates under static conditions using non-buffered C medium at a starting pH of 7.5 (circles) or the same medium at a starting pH of 6.4 (triangles); each dot represents the mean value of three independent experiments performed for each of the 29 isolates.

In subsequent experiments, pH involvement in GAS biofilm formation was evaluated by plate assay using 29 strains belonging to 7 FCT-types. All tested isolates were grown for 12 hours in non-buffered medium without supplemented glucose at a starting pH of 7.5 or 6.4, and the absorbance in each well was measured after crystal violet staining. Results are shown in Figure 3 and mirror those reported in Figure 1. Of note, FCT-1 strains formed biofilm irrespective of the starting pH value, whereas all the remaining strains showed increased biofilm formation when grown in media with lower starting pH conditions, except for M75 (FCT-9), M28 and M89 (FCT-4) strains, which were confirmed as poor biofilm formers. Overall, the data indicate that environmental acidification to pH values lower than 5.3 acts as a signal for the majority of GAS isolates belonging to FCT-types 2, 3, 4, 5 and 6 to start biofilm formation.

### The capacity of an FCT-3 isolate to form microcolonies on epithelial cells is dependent on pH

The association between low pH and biofilm formation on abiotic surfaces was confirmed by confocal microscopy analysis of the FCT-3 strain 43_M3 grown on glass slides in non- buffered low glucose medium at pH 6.4 or 7.5 (Figure S1 A). As shown, bacteria grown at pH 7.5 adhered poorly to the coverslip and did not form any biofilm, while bacteria grown at a starting pH of 6.4 formed large three-dimensional structures.

The higher capacity of the 43_M3 isolate to form biofilm on plates at low pH paralleled its higher tendency to form aggregates in suspension. Bacteria were grown as standing cultures using non-buffered C media either at pH 7.5 or 6.4 and optical density was followed over time. As shown in Figure S1 B, optical density values at the two different growth conditions increased in parallel up to optical density at 600 nm (OD_600_) of 0.6 (early stationary phase), when bacteria at low pH started to precipitate towards the bottom of the tube, resulting in a sequential OD_600_ decrease. Figure S1 C shows a picture of the standing cultures after 12 hour incubation, where precipitation of the bacteria grown at starting pH of 6.4 can be observed.

We subsequently investigated whether low pH drives the formation of large three-dimensional microcolonies during adhesion of GAS 43_M3 to host cells. The microorganisms were grown in DMEM enriched medium either at pH 6.4 or 7.5 up to OD_600_ 0.3, when the bacterial cultures and their growth medium (pH 6.2 and 6.9 respectively) were transferred onto monolayers of pulmonary epithelial cells.

**Figure 4:**
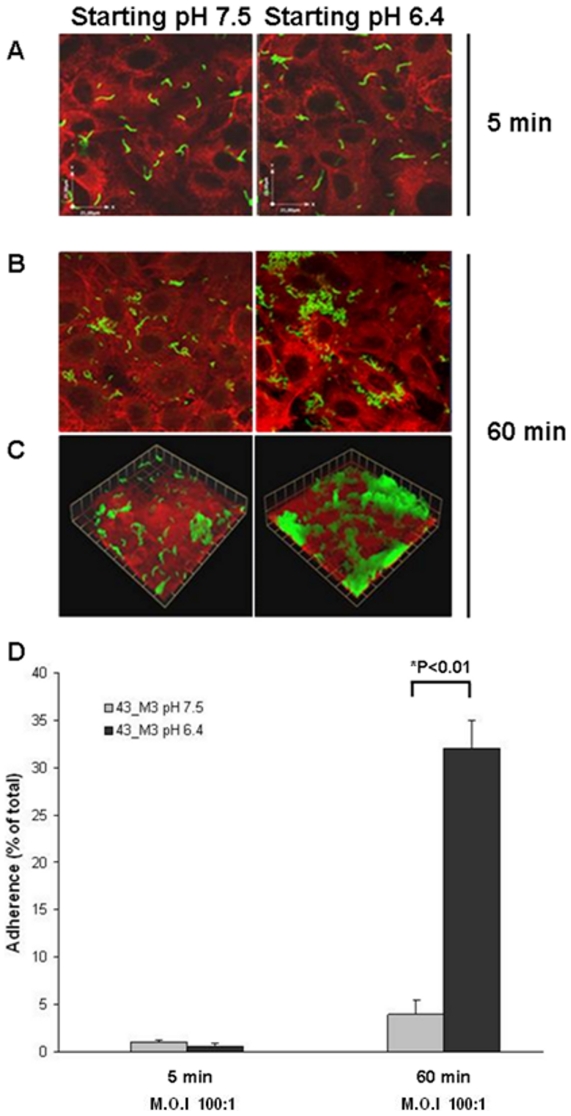
Effect of pH on adhesion of strain 43_M3 (FCT-3) to the A549 lung epithelial cell line. Bacteria were grown to exponential phase (OD_600_  = 0.3) in non-buffered DMEM enriched medium either at pH 6.4 or 7.5; the two bacterial cultures were then used to infect cells, at a M.O.I of 100: 1, up to 5 and 60 minutes. **A–C**) Immunofluorescence analysis (magnification 60 x) of 43_M3 (FCT-3) adhering to A549 cells after 5 and 60 minutes of co-incubation, followed by staining with anti-GAS mouse sera and secondary antibodies conjugated with Alexa Fluor 488 (green); eukaryotic cells were stained with phalloidin-conjugated Alexa Fluor 647 (red); **A**) Image obtained after 5 minutes of infection. **B**) Image obtained after 60 minutes of infection. **C**) Three-dimensional reconstructions 60 minutes after infection. **D**) Percent of total inoculated bacteria associated to epithelial cells after 5 and 60 minutes of incubation, extensive washing, cell lysis and sonication; results are presented as mean and standard deviation values of three independent-experiments.

Confocal microscopy analysis after 5 minutes of incubation and three consecutive washings revealed small chains, irrespective of the culture medium used for growth (panel A of Figure 4). After 60 minutes, pH values had shifted from 6.2 to 5.8 and from 6.9 to 6.7, and cell adhering bacteria appeared as microcolonies, which were of superior dimension at lower pH as compared to higher pH (Figure 4, panel B). Accordingly, bacterial three-dimensional multilayered structures were observed only in the case of bacteria grown at low pH (Figure 4, panel C). Control experiments revealed that membrane integrity at the investigated time points was not affected by pH (data not shown). Longer incubation times were not investigated to avoid any possible cell membrane integrity disruption due to low pH.

In line with the above observations, the number of adhering colony forming units at the 60- minute time point, measured after extensive washing of the plates followed by bacterial sonication and plating, was 10-fold higher when bacteria pre-cultured at starting pH of 6.4 were used for cell infection as compared to pH 7.5 pre-cultures (panel D). Bacterial growth during cell infection, calculated by adding the number of microorganisms present in the supernatants to the number of cell-adhering bacteria, was similar at the two different pH values.

Altogether, the data indicate that environmental acidification acts as a signal on the FCT-3 isolate 43_M3 to start cell adhesion in the form of large multi cellular biofilm-like communities, resulting in high numbers of bacteria associated to epithelial cells.

### Expression of pili is pH-dependent in FCT-2, FCT-3 and FCT-4, but not in FCT-1 strains

We subsequently asked whether differential pilus expression could be directly involved in the observed FCT-type specific biofilm response to pH stimulation. To this aim, four high biofilm former strains belonging to variants FCT-1 (M6), FCT-2 (M1), FCT-3 (M3) and FCT-4 (M12) were grown in non-buffered medium at pH 7.5 or 6.4, and the relative amounts of one of the pilus proteins at the two pH growth conditions was analyzed by Western Blot. Equivalent quantities of cell wall-enriched extracts from each strain grown at pH 7.5 or 6.4 were loaded onto SDS PAGE, transferred to nitrocellulose membranes and analyzed by mouse sera specific for the ancillary protein 1 (AP1) variant of each pilus type. As an internal control, membranes were incubated with antibodies recognizing the GAS surface protein Spy0269, which was found to be equally well expressed at pH 7.5 or 6.4 by real-time reverse transcription PCR (RT-PCR, data not shown). The same nitrocellulose membranes were exposed to antibodies against the 4 M-protein variants, as this important GAS virulence factor has also been shown to play a role in biofilm formation [10].

**Figure 5:**
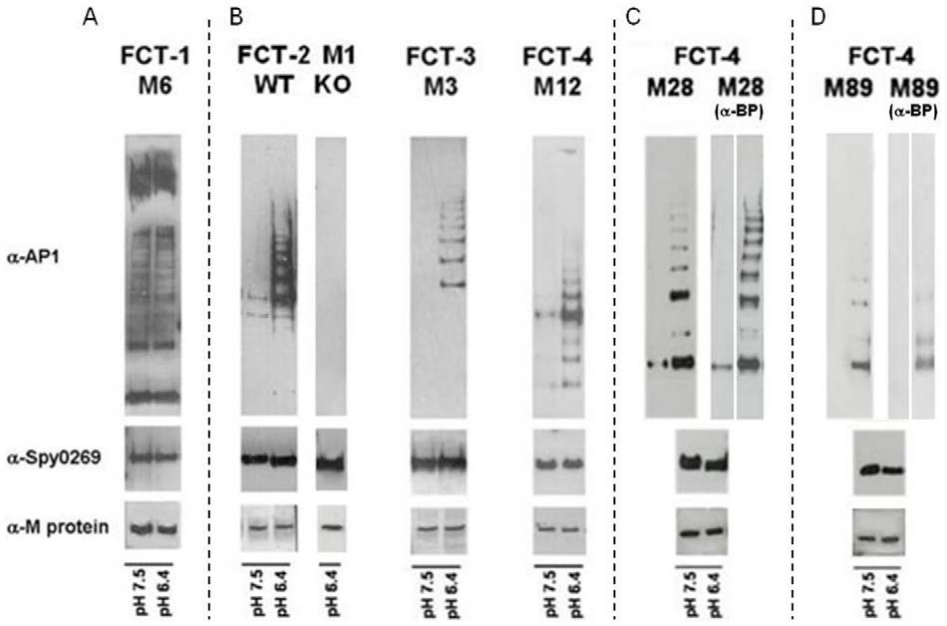
Expression of pili in FCT-1, FCT-2, FCT-3 and FCT-4 isolates grown at different pH conditions. Immunoblot analysis of cell surface extracts of GAS strains 27_M6 (FCT-1), 51_M1 (FCT-2), 43_M3 (FCT-3), 135_M12 (FCT-4) 57_M28 (FCT-4) and 96_M89 (FCT-4) grown in non-buffered C-medium at starting pH values of 7.5 or 6.4 up to OD_600_ of 0.4; nitrocellulose transferred extracts were incubated with specific mouse polyclonal sera raised against M protein variants,Spy0269 protein, specific AP-1 pilin proteins and, in the case of the non biofilm formers M28 and M89, specific AP-1 and BP proteins.

As shown in Figure 5A, the characteristic ladder corresponding to pilus polymeric structures was highly and equally evident in GAS M6 FCT-1 extracts at both pH conditions. Conversely, FCT-2, FCT-3 and FCT-4 pilus polymers were barely detectable at pH 7.5, whereas their presence was found to be enhanced at pH 6.4 (panel B in Figure 5). Moreover, neither the concentration of M protein or Spy0269 was affected by pH in any of the four strains.

To investigate whether the incapacity to form biofilm of the FCT-4 subgroup of isolates belonging to M types M28 and M89 correlated with a lack of pilus expression at any pH condition, we analyzed by Western Blot cell wall-enriched extracts of four M28 and four M89 strains using sera specific to the corresponding AP1 and backbone (BP) pilus variants. The results indicated that all four M28 strains expressed pili to a similar extent as FCT-2 M1, FCT-3 M3 and FCT-4 M12 isolates and, also in this case, pilus expression was enhanced in cultures at starting pH of 6.4 compared to those at 7.5. Conversely, AP1 and BP expression in the four analyzed M89 strains was much lower, even if it slightly increased at lower pH. Immunoblot analyses of one representative strain for each of the two M28 and M89 types are shown in Figure 5 panels C and D respectively. Overall, the data indicate that expression of pili is pH-dependent in FCT-2, FCT-3 and FCT-4, but not in FCT-1 strains.

Therefore, four types of GAS isolates were identified based on the effect of environmental pH on biofilm formation and on pilus expression: (I) pH independent biofilm formers, expressing pili in a pH independent manner (belonging to the FCT-1 type); (II) pH-dependent biofilm formers, all of which showing pH-dependent pilus expression (FCT-2 FCT-3, FCT-5 and FCT-6, plus a subset of FCT-4 strains belonging to the M12 serotype); (III) non biofilm formers expressing pili in a pH-dependent manner (like the FCT-4 M28 isolates); (IV) non biofilm formers showing very low pH-dependent pilus expression (like the FCT-4 M89 isolates).

### Several FCT-2 and FCT-3 genes are differentially transcribed according to pH

To investigate whether differences in the amount of pilus proteins observed for FCT-2 and FCT-3 strains correlates with pH-dependent mRNA levels, we performed real-time reverse transcription PCR (RT-PCR) experiments to compare the relative amounts of the pilus AP-1 transcripts in the previously investigated FCT-1, FCT-2 and FCT-3 strains, following bacterial growth either at pH 6.4 or 7.5.

**Figure 6:**
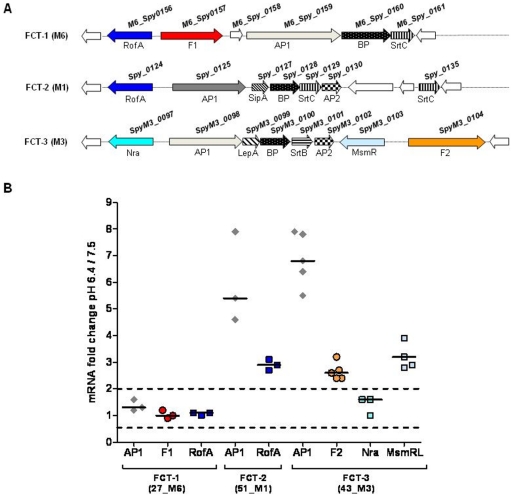
Transcript levels of FCT-1, FCT-2 and FCT-3 genes at different pH growth conditions. **A**) Genetic organization of FCT-1, FCT-2 and FCT-3 regions. Transcription regulators are in blue, genes of the pilus operons in black-grey, and F proteins in red-orange. **B**) Relative transcript abundance of FCT encoded genes in strains 27_M6 (FCT-1), 51_M1 (FCT-2) and 43_M3 (FCT-3) grown up to OD_600_ of 0.4 in non-buffered C-medium at starting pH of 6.4 or 7.5 (gene colors match those of panel A); transcript abundance was measured using the housekeeping gene *gyrA* as a control; data are reported as mean value of at least three independent experiments performed with independently isolated RNA samples.

As shown in figure 6A, these FCT regions contain genes encoding the pilus components (the backbone protein BP and the ancillary proteins AP1 and AP2), and the pilus assembly machinery (which comprises sortases SRT, and the proteins SipA and LepA, displaying homology with signal peptidases). Other genes present in the FCT regions encode F1 and F2 fibronectin-binding adhesins (Figure 6A, red-orange colors), as well as transcription regulators which control the expression of the genes present inside these DNA regions and elsewhere in the GAS genome (Figure 6A, blue colors) [17], [18], [21]. Thus, we also investigated whether the F1 and F2 proteins and the transcription regulators RofA, MsmR and Nra were expressed in a pH-dependent manner. In particular, we measured by RT-PCR the relative mRNA amounts at pH 6.4 or 7.5 of the genes encoding AP1, F1 and RofA in strain 27_M6 (FCT-1), AP1 and RofA in strain 51_M1 (FCT-2), and finally AP1, F2 and the two regulatory proteins MsmR and Nra in strain 43_M3 (FCT-3).

As shown in Figure 6, no significant differences in the quantity of transcript corresponding to AP1 and F1 proteins were detected in the FCT-1 isolate grown either at pH 6.4 or at pH 7.5. On the contrary, transcript amount of the gene encoding AP1 in FCT-2 and of those encoding AP1 and F2 in FCT-3 was enhanced at lower pH levels. Similar results as for the gene encoding AP1 were obtained for other genes involved in pilus assembly, previously reported to be transcribed as a single operon [22], [23] (data not shown).

Concerning regulatory proteins, the RofA message in M6 (FCT-1) was not affected by pH. Conversely, the amount of FCT-2 RofA mRNA was higher at pH 6.4. Furthermore, according to mRNA abundance, expression of the gene encoding the MsmR regulator in the FCT-3 strain [23], [24] was enhanced under acidic conditions, while no pH effect was detected for the Nra FCT-3 regulator gene.

The data suggest that regulation of pili and/or protein F2 expression by pH in FCT-types 2 and 3, could be directly associated to the higher mRNA amounts of RofA and MsmR regulators at low environmental pH.

### pH-dependent biofilm formation is directly associated with differential pilus expression

Having demonstrated that in the FCT-3 analyzed strain the expression of pili and proteins F1 and F2 is enhanced under acidic environments, we next investigated whether differential expression of any of these gene products was directly involved in pH-dependent biofilm formation.

With this aim, we first asked whether constitutive expression of pili in the FCT-3 43_M3 strain could result in the formation of biofilm irrespective of pH. A DNA fragment from the FCT region of 43_M3 comprising all genes involved in pilus expression, i.e. *SpyM3_0098* (AP1), *SpyM3_ 0099* (signal peptidase), *SpyM3_0100* (backbone protein), *SpyM3_0101* (sortase) and *SpyM3_0102* (AP2), was inserted into a plasmid vector under the control of a heterologous promoter known to drive gene expression in GAS [25], [26]. The whole operon with the original self-promoter region was inserted in the same vector as control. The two resulting plasmids pAM(self-pilusM3) and pAM(p80-pilusM3) were introduced into the 43_M3 strain and surface protein extracts from recombinant clones were analyzed by Western blot using AP-1 specific antibodies.

**Figure 7:**
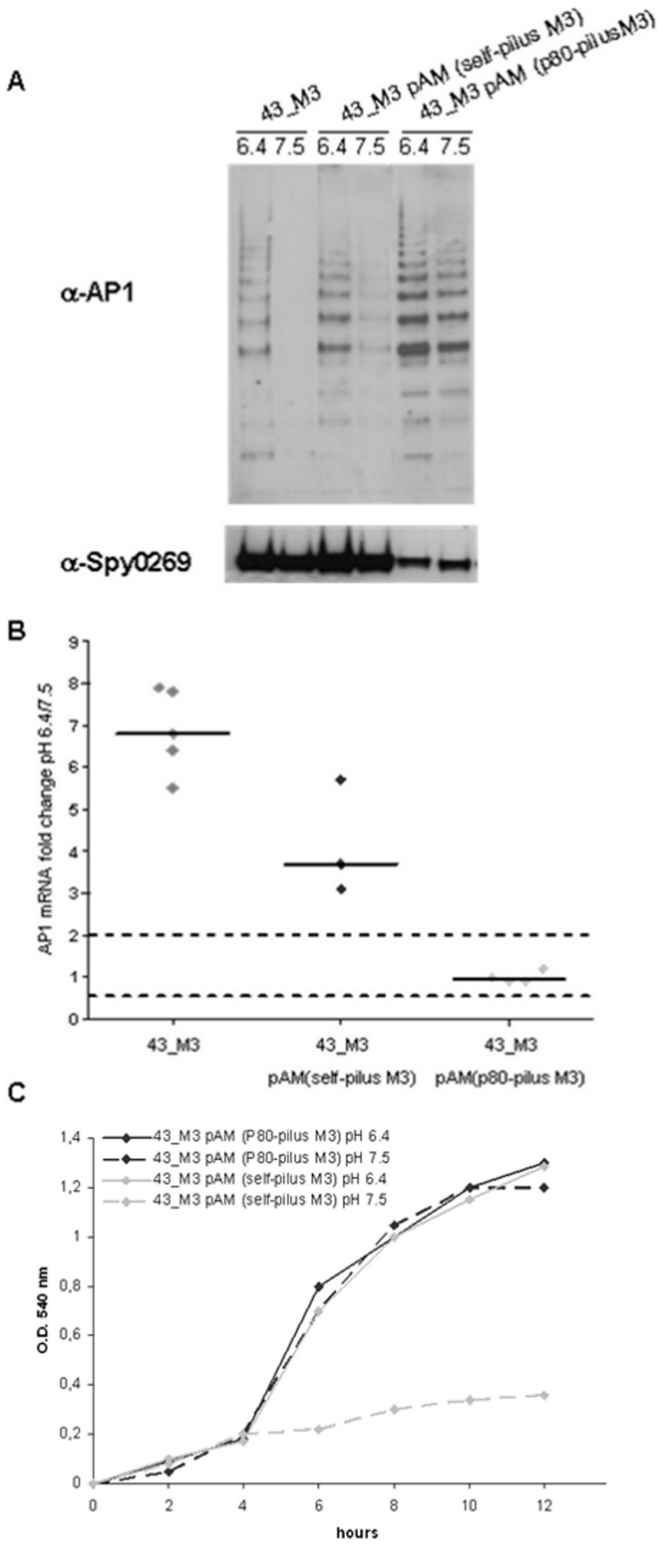
pH-independent pilus expression in 43_M3 (FCT-3) recombinant derivatives and its effect on biofilm formation. Wild-type 43_M3 (FCT-3) and recombinant derivatives “43_M3 pAM (self-pilusM3)” (over-expressing pilus M3 under its natural self-promoter) or “43_M3 pAM (p80-pilusM3)” (heterologous constitutive promoter) were grown in non-buffered C-medium at starting pH of 7.5 or 6.4. **A**) Cell surface enriched extracts of wild-type and recombinant bacteria grown to OD_600_ of 0.4 were loaded onto SDS-PAGE, transferred to nitrocellulose and incubated with mouse polyclonal sera raised against AP-1 pilus protein from strain 43_M3 (FCT-3) or against protein Spy0269 protein (control). **B**) Comparison of *ap-1* gene transcription in wild-type 43_M3 (FCT-3), “43_M3 pAM (self-pilusM3)”, and “43_M3 pAM (p80-pilusM3)” grown at the two pH conditions up to OD_600_  = 0.4. **C**) Time-course biofilm plate assays using “43_M3 pAM (self-pilusM3)” (black) and “43_M3 pAM (p80-pilusM3)” (grey) bacteria grown in C medium at a starting pH of 7.5 (dotted lines) or 6.4 (full lines).

As expected, pilus expression was dependent on pH in bacteria harboring the DNA pilus region under its self-promoter, while high constitutive expression was detected for recombinant bacteria carrying the heterologous promoter (Figure 7A). The data were confirmed by RT-PCR (Figure 7B).

Remarkably, as shown in Figure 7C, the 43_M3 strain derivative constitutively expressing FCT-3 pili acquired the capacity to form biofilm independently of environmental pH, while expression of the FCT-3 pilus operon under its own promoter resulted in pH-dependent biofilm formation, as previously observed for the original 43_M3 strain. Overall, the data indicate that pH-driven biofilm formation in the FCT-3 43_M3 strain, and possibly in all other pH-dependent strains, relies on differential pilus expression.

**Figure 8:**
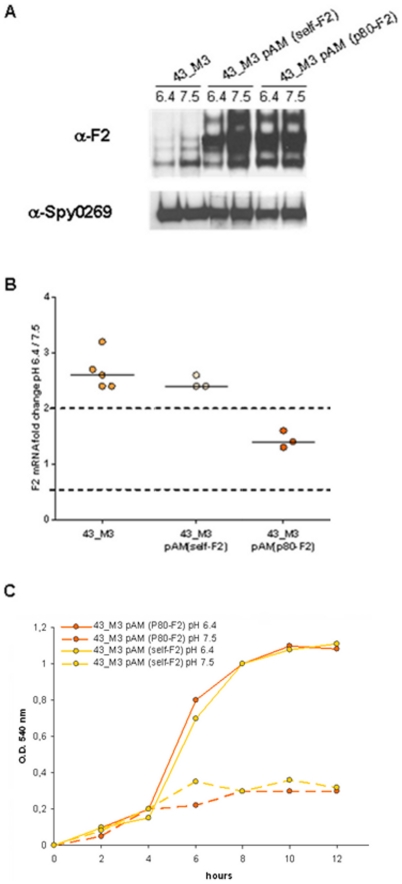
pH-independent expression of protein F2 in 43_M3 (FCT-3) recombinant strain derivatives and its effect on biofilm formation. Wild-type 43_M3 (FCT-3) and recombinant derivatives “43_M3 pAM (self-F2)” (over-expressing protein F2 under its natural self-promoter) or “43_M3 pAM (p80-F2)” (heterologous constitutive promoter) were grown in non-buffered C-medium at starting pH of 7.5 or 6.4. A) Cell surface extracts of wild-type and recombinant bacteria grown to OD_600_ of 0.4 were loaded onto SDS-PAGE, transferred to nitrocellulose and incubated with mouse polyclonal sera raised against protein F2 from strain 43_M3 (FCT-3) or against Spy0269 protein (control). B) Comparison of *prtf2* gene transcription in wild-type 43_M3 (FCT-3), “43_M3 pAM (self-pilusM3)”, and “43_M3 pAM (p80-pilusM3)” grown to OD_600_  = 0.4 using the two distinct pH conditions. C) Time-course biofilm plate assay using “43_M3 pAM (self-F2)” (yellow) and “43_M3 pAM (p80-F2)” (orange) bacteria grown in C medium at a starting pH of 7.5 (dotted lines) or 6.4 (full lines).

As mentioned above, a second protein possibly involved in pH-dependent biofilm formation in the FCT-3 43_M3 strain was the fibronectin-binding protein F2, since its expression was also enhanced at low pH. To investigate the effect of constitutive expression of protein F2 on biofilm, we performed similar experiments as those reported for the pilus region. In particular, the gene encoding protein F2 in strain 43_M3 was episomally introduced into the same strain both under the control of the natural self-promoter and of a heterologous promoter. Plasmids pAM(self-F2) and pAM(p80-F2) were transformed into 43_M3, and surface protein extracts from recombinant clones were analyzed by Western blot using protein F2- specific antibodies. As shown in Figure 8A and 8B, 43_M3 containing pAM(self-F2) over expressed protein F2 in a pH-dependent manner, while expression became constitutive in clones harboring the plasmid pAM(p80-F2). Recombinant 43_M3 bacteria were then tested for their capacity to form biofilm at different starting pH. Interestingly, both clones produced biofilm only at low pH (Figure 8C), indicating that differential expression of protein F2 has no effect on pH-dependent 43_M3 biofilm formation.

Taken together, the data show that pH-dependent GAS biofilm formation is directly related to pilus expression but not to F2 protein expression.

## Discussion

The *S. pyogenes* FCT genomic region, first described as encoding several transcription regulators, variable fibronectin and collagen binding adhesion molecules, as well as the T antigen used for GAS serotyping [16], [27], stimulated the interest of the GAS scientific community after it was shown to be responsible for the formation of pilus-like appendages protruding outside the GAS surface that can be used as vaccine targets [28]. We recently reported that GAS pili are involved in cell adhesion and in biofilm formation [14], while other authors showed that they may play a role in the establishment of skin infections [29].

In the present study we investigated the possible relationship between the variability of the FCT region and the capacity of different GAS strains to build biofilm-like microbial communities in different culture conditions. By analyzing a collection of clinical isolates belonging to 7 different FCT variants for their capacity to form biofilm on microplate polystyrene surfaces, we observed that all FCT-1 strains formed large microbial communities on abiotic surfaces independently of the presence of glucose in the growth medium, while isolates belonging to other FCT types required glucose or other sugars to form biofilms. The increased biofilm forming capacity in the presence of 30 mM glucose was found to be a consequence of the environmental acidification resulting from sugar consumption during bacterial growth, as a similar FCT-dependent biofilm phenotype could be achieved by lowering the starting pH of the medium without any sugar supplementation, but not in buffered medium containing 30 mM glucose.

It has recently been suggested that members of the M protein family mediate anchoring of lipoteichoic acids to the surface of *S. pyogenes*, leading to increased hydrophobicity and enhanced biofilm formation by the pathogen [30]. We observed that pH-dependent biofilm formation did not rely on increased bacterial hydrophobicity at low pH, as comparable adherence to hexadecane droplets was obtained for bacteria grown at different starting pH conditions (data not shown).

We hypothesize that modulation of pilus expression and biofilm formation in response to pH could play an important role during the initial cell adhesion steps of GAS infection, as suggested by the results obtained when bacteria belonging to the pH-dependent FCT-type 3 pre-inoculated either at a starting pH of 6.4 or 7.5 were co-incubated with epithelial cell monolayers. Bacteria cultured at lower starting pH formed large three-dimensional microcolonies and, probably as a consequence of this, the number of cell-adhering microorganisms after 1 hour of co-incubation was 10 times higher in the case of bacteria derived from pH 6.4 inocula compared to bacteria grown at starting pH of 7.5.

A role for pH in host colonization and infection by Gram positive pathogens including GAS, was previously proposed based on three main lines of evidence. First, the diverse environments encountered by these microorganisms are characterized by local and temporal pH fluctuations. Particularly, pH varies in three relevant niches for GAS colonization and infection initiation: in the oral cavity from 5 to 7 [31], in the naso-pharynx from 6.4 to 6.9 [32] and on the skin from 4.2 to 5.9 [33], [34]. Moreover, pH changes in the skin barrier are associated with conditions such as atopic dermatitis [35] and may also occur during the formation of abscesses and skin necrotic lesions [36].

Second, bacterial growth can cause local pH alterations, which can subsequently be sensed by some organisms as a signal to modify their growth mode. For instance, it has been shown that in the oral cavity a pH change associated with sugar metabolism by dental pathogens results in a switch from a healthy status to disease, which parallels the formation of mature multi-species biofilms [37], [38], [39]. Moreover, metabolism of glucose and other sugars by *S. aureus* leads to a pH decrease of the culture [40], [41], which has been shown to be associated with the accumulation of acetic and other organic acids [42]. Interestingly, it was recently reported that in MRSA *S. aureus* strains, biofilm development is promoted under mildly acidic growth conditions triggered by the addition of glucose to the growth medium [43]. In this instance, it is tempting to speculate that local pH lowering resulting from sugar consumption on the cell surface may drive the formation of firmly adhering biofilm-like microbial communities.

Third, many Gram positive pathogens modulate gene expression in response to pH changes. For instance, *S. mutans* survives and proliferates at low pH by up-regulating a number of genes that protect against acid stress [44]. Moreover, microarray analysis revealed a differential expression of approximately 10 to 15% of the total number of genes in *Staphylococcus aureus* [41], *S. pyogenes* [20] and *S. agalactiae* [45] under mildly acidic conditions compared to neutral pH. Of note, no differences in pilus expression were detected in the *S. pyogenes* microarray, probably because the growth conditions investigated in that study (non-buffered medium at pH 7.5 or buffered medium at pH 6) did not lead to attain pH values under 5.4, as in our experiments.

Interestingly, gene expression regulation at low versus neutral pH has been show to correlate with differential expression during exponential-phase planktonic cultures versus mature biofilm in *S. aureus* [39] and, in the case of *S. pyogenes*, with differential gene expression during murine subcutaneous infection versus *in vitro* growth at neutral pH [20].

The finding that biofilm formation was pH-dependent in some but not all GAS FCT-types, led us to further investigate whether this phenotype variation was associated with a difference in the expression of FCT-encoded genes. Indeed, Western Blot and RT-PCR experiments revealed higher amounts of pilus proteins and fibronectin-binding adhesins in FCT-2, FCT-3 and FCT-4 M12 isolates when bacteria were grown at a starting pH of 6.4 as compared to growth at pH 7.5. In contrast, pH-independent pilus expression was observed in the case of an FCT-1 strain. Concerning pilus expression in non biofilm former strains, we observed pH-dependent expression in FCT-4 M28 as well as in M89 strains (which, however, expressed pili at a much lower extent). The data suggest that M28 strains may lack a still undefined factor required, in addition to the pili, for biofilm formation. The same could be true for M89 strains, although in this case the incapacity to form biofilm could also be associated to low pilus expression. Therefore, pili appear to be necessary but not sufficient for GAS biofilm formation, confirming previous literature data indicating that this mode of growth relies on multiple bacterial components.

Finally, constitutive expression of GAS pili in a FCT-3 recombinant strain resulted in pH-independent biofilm formation, in contrast to the results obtained with a recombinant strain constitutively expressing the F2 protein. This confirmed GAS pili and not F2 proteins to be directly involved in pH-dependent auto-aggregation and biofilm formation on biotic and abiotic surfaces.

Besides genes encoding fibronectin-binding adhesins and the pilus machinery, all FCT genomic regions contain genes coding for stand alone transcription regulators. These regulators, the RofA/Nra homologues and MsmR, coordinately control the expression of the FCT open reading frames and of important virulence factors outside this region, during different bacterial growth phases [22], [23]. Further on, they are involved in the response to changes of environmental parameters like oxygen pressure [23], [46], [47] and temperature [48].

Our results indicate that the expression of the FCT transcription regulators is affected by pH. In fact, in the analyzed FCT-3 M3 strain, *msmR* transcript amounts increased at lower pH, in parallel to the enhanced expression of pili and the F2 protein. MsmR and Nra participate in the fine tuning of FCT-3 pilus expression by exerting their function in opposite directions and have been shown to act either as positive or negative regulators, depending on the strain genetic background [23], [24], [49]. In particular, in a FCT-3 M49 strain, expression of pili and the F2 protein were shown to be repressed by Nra and activated by MsmR [22], [23]. Conversely, in a FCT-3 M53 isolate, Nra acted as an activator of pilus expression without affecting F2 expression, while MsmR repressed pilus transcription and activated the expression of F2 [24].

Our data showing that in 43_M3 both MsmR and pili were more expressed at lower pH, suggest that in this strain MsmR exerts a positive effect on pilus transcription. Despite previous observations indicating that the Nra regulator is under the control of MsmR [23], we detected only a mild difference (1.6 fold in 2 out of 3 experiments) in the amount of *nra* transcripts at the two investigated pH conditions.

Increased transcript amounts at acidic pH conditions were also detected for the FCT-2 pilus positive regulator RofA in strain 51_M1. Interestingly, a copy of *rofA* is also present in the FCT-1 region, which exerts a positive effect on the expression of the F1 protein gene, of the pilus operon and other targets located elsewhere in the genome [27], [47], [50]. However, despite the fact that FCT-1 and FCT-2 *rofA* genes and their intergenic regions share 98% identity, the FCT-1 variant was highly and constitutively transcribed irrespective of environmental pH. These results recall those obtained by Granok and collaborators [47], who showed high transcription rates of the genes located downstream *rofA* in a GAS M6 (FCT-1) strain both under aerobic and anaerobic conditions, while in M1 and M5 strains strong expression was exclusively observed under aerobic conditions. Interestingly, when the M6 RofA/F1 intergenic region carrying a downstream reporter gene was transferred into M1 or M5 strains, it drove gene expression only under aerobic conditions. Based on those results, the authors postulated that independent pathways in addition to RofA could be involved in F1 activation.

Our data suggest that in FCT-2 and FCT-3 strains environmental pH could be sensed by a so far unknown factor which affects the transcription of MsmR and RofA regulators and consequently, of their controlled genes. Of note, sensor kinase components of various two-component systems (TCS) have been reported as acidic pH sensors in several Gram negative bacteria [51], [52], [53]. Furthermore, a direct negative regulation of the *S. pneumoniae* pilus rlrA regulator and indirectly of its pilus subunits by the two component systems TCS03 and TCS06 has been reported [54].

Among the GAS TCS, CovRS appeared as a possible candidate directly responding to pH variations. Indeed, Dalton and Scott [55] showed that GAS growth at low pH was impaired in a CovS knock-out mutant strain. Moreover, Santi and co-workers [45] recently reported that a CovRS homologue in *S. agalactiae* was in part involved in pH-dependent gene expression regulation. To investigate this possibility, in preliminary experiments we compared by Western Blot the amount of pilus proteins expressed at starting pH conditions of 6.4 and 7.5, using an FCT-4 M12 strain and its mouse passaged derivative containing a stop codon in the middle of the CovS gene. As shown in Figure S2, pilus proteins were expressed at a similar extent and in a pH-dependent manner by the two strains, suggesting that this TCS is not directly involved in pH-dependent expression of the FCT-4 genes. The data were further confirmed assessing pH-dependent pilus protein expression in a FCT-2 M1 CovS mutant (Figure S2).

In conclusion, the results here reported indicate that most GAS FCT-types sense environmental pH as a signal to build pilus appendages on their surface, and suggest that this process leads to the formation of large cell-adhering multicellular communities which may assist the initial steps of GAS infection.

## Materials and Methods

### Bacterial strains and growth conditions

The 44 *S. pyogenes* clinical isolates analyzed in this paper were obtained in the University Hospital of Rostock (Germany) during the time period from 2001 to 2006 [19]. The presence or absence of FCT-specific gene products was assessed by PCR, using the genomic DNA of each strain as template, and the obtained gene patterns were assigned to established FCT-types as described [18], [19]. For maintenance, GAS isolates were incubated on THY agar plates supplemented with 5% defibrinated sheep blood. For biofilm experiments *S. pyogenes* isolates were grown at 37°C in C medium [56] modified as follows: a) adjusted to pH 7.5 with NaOH; b) adjusted to pH 7.5 with NaOH and supplemented with 30 mM glucose; c) buffered using 100 mM Na_2_HPO_4_/NaH_2_PO_4_ at pH 7.5 and supplemented with 30 mM glucose; d) adjusted to pH 6.4 with HCl. For cell adhesion experiments we used Dulbecco's modified Eagle's medium (DMEM) depleted of red phenol, sodium carbonate, sodium phosphate and glucose and supplemented with 0.5% proteose peptone #3 and 1.5% yeast extract (DMEM-b).

*Escherichia coli* were grown in Luria Broth. Selective media contained 10 µg/ml and 20 µg/ml Chloramphenicol for GAS and for *E. coli*, respectively.

### Biofilm plate assays

For each strain, an overnight culture (C-medium, 37°C) was diluted 1∶20 in fresh C medium (modified as mentioned above) and 1 ml was added to each well of a 24-well plate. Plates were incubated for different time periods at 37°C. Subsequently the medium was removed, wells were washed with PBS (phosphate buffered saline), and adherent bacteria were stained with crystal violet (0.2% in ddH_2_O, room temperature, 10 min). After washing with PBS, crystal violet was recovered with 1% SDS and the biomass was quantified by measuring the optical density at 540 nm (OD_540_). Analyses were performed in triplicate in three distinct experiments and mean results are presented.

### Hydrophobicity assay

Strain 43_M3 (FCT-3) was cultured in non-buffered C medium at pH 7.5 or pH 6.4. Ten ml cultures were centrifuged, washed in PBS and adjusted to an optical density at 600 nm OD_600_  = 1 in PBS. One ml of bacteria was added with 300 µl of hexadecane (Fluka-BioChemiKa) and vortexed for 15 seconds. Samples were incubated for 30 minutes at room temperature and the optical density of the aqueous phase was measured at 600 nm. Relative sample hydrophobicity was calculated as 1-OD_600_ H_2_O phase/1, assuming 100% as OD_600_  = 1.

### Confocal microscopy (CLSM) analysis of biofilms on abiotic surfaces

Group A *Streptococcus* strains were grown overnight in C-medium at 37°C diluted 1∶20 in fresh C medium adjusted to pH 7.5 or 6.4 and thereof and 1 ml was inoculated at room temperature on uncoated glass sterile coverslips positioned in a 12-well plates. Samples were grown for 10 hours at 37°C, fixed, blocked and stained with rabbit-anti-GAS and with Alexa Fluor dye 488 goat anti-rabbit as secondary antibody (Molecular Probes) according to manufacturer's protocol. Coverslips were then washed with blocking solution, mounted on glass slides with the Slow Fade reagent kit containing 4.6-diamidino-2-phenylindole dihydrochloride (Molecular Probes) and viewed on a Bio-Rad Radiance 2000 Scanning Laser Confocal Microscope. Three-dimensional immunofluorescence images were reconstructed from 0.5 mm confocal optical sections using VOLOCITY 3.6 software (Improvision, Lexington, MA, USA).

### Determination of bacterial aggregation

Strain 43_M3 (FCT-3) was grown in 10 ml tubes to stationary phase in C medium at pH 7.5 or 6.4 without agitation. The precipitation rate was determined by measuring light absorbance at OD_600_ in the upper part of the tubes at regular time intervals. GAS aggregation was also observed using optical light microscopy. Exponential phase bacterial pre-cultures were seeded in 12-well plates containing sterile glass coverslips and incubated in C medium at pH 7.5 or 6.4 without agitation, to attain OD_600_ values of 0.3 and 0.6. Samples were fixed with formaldehyde 2% (Sigma-Aldrich, St. Louis, MO, USA) in PBS for 15 min, washed with PBS and stained with 0.2% crystal violet for 10 minutes. Samples were then washed in PBS and imaged on an Axiovert 40 CFL Optic Light Microscope (Carl Zeiss Ag, Germany) equipped with a Canon powershot A640 camera.

### Eukaryotic cell cultures and adherence assays

The human lung adenocarcinoma epithelial cell line A549 (ATCC CCL-185) was cultured in Dulbecco's modified Eagle's medium (DMEM; Gibco BRL, Invitrogen Life Technologies, USA) supplemented with 10% FCS (Gibco BRL, Invitrogen Life Technologies, USA) and 5 mM glutamine (Gibco BRL, Invitrogen Life Technologies, USA) at 37°C in an atmosphere containing 5% CO_2_. For adherence assays, cells were resuspended at a concentration of approximately 5×10^5^ cells ml^−1^ in DMEM, and 1 ml seeded into 12 well tissue culture plates, which were then incubated for 24 h. For time-course experiments bacteria were grown in DMEM-b at starting pH 7.5 and 6.4. At early exponential phase (OD_600_  = 0.3), bacteria in their growth media were used to infect cell monolayers at 37°C in a 5% CO_2_ atmosphere using a multiplicity of infection (M.O.I) of 100∶1. After different infection times, wells were extensively washed with PBS to remove unattached bacteria and were either used for staining and confocal microscopy (CLSM) or for counting the number of cell-adhering colony forming units (CFU).

For analysis by CLSM, cells were fixed, blocked and stained with rabbit-anti-GAS and with Alexa Fluor 488-conjugated goat anti-rabbit secondary antibody (Molecular Probes, Invitrogen Life Technologies, USA). A549 cells were stained with Alexa Fluor 647-conjugated phalloidin (Molecular Probes). Coverslip mounting, viewing and three-dimensional reconstructions were performed as described above.

For CFU counting, eukaryotic cells were lysed with 1% saponin and 0.25% trypsin. After sonication (3×10 seconds at 50 Hz, UP50H Hielscher Ultrasonics, Germany) cell-adherent bacteria were plated for enumeration. The average number of bacteria recovered per ml was determined from three independent wells. Tests were repeated at least three times and the percentage of adhering bacteria versus total bacteria was calculated for each time point.

To evaluate cell membrane integrity, A549 cells were incubated for 20 minutes with live\dead reagent (L34960 Molecular Probes, Invitrogen Life Technologies, USA) washed four times with PBS and analyzed by CLSM.

### Immunoblots of bacterial cell-wall fractions

Bacterial cell-wall enriched fractions were prepared as previously described [28]. Briefly, bacteria grown in non-buffered C medium either at a starting pH of 7.5 or at pH 6.4 to log phase (OD_600_  = 0.4) were pelletted, washed once in PBS, suspended in 1 ml ice-cold protoplasting buffer [40% sucrose; 0.1 M KPO_4_, pH 6.2; 10 mM MgCl_2_; EDTA-free protease inhibitors (Roche Applied Science, Germany); 2 mg/ml lysozyme (Sigma-Aldrich, St. Louis, MO, USA); 400 units of mutanolysin (Sigma-Aldrich, St. Louis, MO, USA)] and incubated at 37°C for 3 h. After centrifugation at 13,000 g for 15 min, the supernatants (cell wall enriched fractions) were separated by 3-8% gradient SDS-PAGE (NuPAGE Tris-acetate gels, Invitrogen Life Technologies) and transferred to nitrocellulose membranes (Whatman, GE, USA). Immunoblot analyses were performed by incubating the membranes with mouse polyclonal antisera against AP1 variants, M protein variants or Spy0269 [28] at a 1∶1000 dilution, secondary antibody (HRP, horseradish peroxidase-linked anti-mouse IgG, DakoCytomation, Denmark) at a 1∶5000 dilution and developed with ECL enhanced chemiluminescence detection substrate (Super-Signal West Pico, Pierce, USA).

### Episomal introduction of the FCT-3 pilus region and of the gene encoding protein F2 into GAS strain 43_M3 (FCT-3)

The genomic region between the Ribosome Binding Site of gene *spyM3*_0098 and the stop codon of *spyM3*_0102 was amplified using primers 1 and 2 (F P80 pil M3 GCTCGCGGCCGCTTGCAAAAGAGGGATAAAACCAAT and R P80 pil M3 GCTCGG ATCCGACTATCTTGCTAAATACCGAG), which allowed the introduction of NotI and BglII restriction sites. The PCR product was cloned into a shuttle vector carrying the p80 promoter and terminator regions of GBS adhesin island-1 [25] to obtain “pAM(p80-pilusM3)”. Moreover, the same DNA fragment with its self-promoter region was amplified by using primers 3 and 4 (F Self pil M3 GCTCGGATCCTATCTCTAATAGACTG TTCAAGATATG and R Self pil M3 GCTCGGATCCGACTATCTTGCTAAATACCGAG) and inserted in the same vector, originating “pAM(self-pilusM3)”.

The gene encoding protein F2 in strain 43_M3 (FCT-3) was episomally introduced into the same strain both under the control of its self-promoter, through plasmid “pAM(self-F2)”, constructed by using primers 5 and 6 (F Self F2 GCTCGTCGACCTCTGATCATAAG ATGTAGACTTGACA and R Self F2 GCTCGGATCCAACCGAGACGATCGATTCCAGA) and of the p80 promoter, through plasmid “pAM(p80-F2)”, constructed by using primers 7 and 8 (F P80 F2 GCTCGCGGCCG CCTCTGATCATAAGATGTAGACTTGACA and R P80 F2 GCTCGGATCCAACCG AGACGATCGATTCCAGA).

The constructs were first obtained in *E. coli* and then used to transform GAS 43_M3 (FCT-3) electro competent cells. Chloramphenicol-resistant colonies were screened by PCR and the selected colonies were then analyzed by Western blot, RT-PCR and in biofilm plate assays.

### RNA isolation and quantitative real-time reverse transcription PCR (qRT-PCR)

GAS 43_M3 (FCT-3) and its recombinant derivatives were grown in non-buffered C-medium cultures either at pH 7.5 or at pH 6.4. During log phase (OD_600_  = 0.4) bacteria were harvested by centrifugation at 3,500 g for 5 min at 4°C, resuspended in 500 µl PBS and incubated for 5 min at RT with 1 ml of Protect (Qiagen, Hilden, Germany) for RNA stabilization. After centrifuging at 6,500 g for 15 min, bacteria were resuspended in 100 µl lysis buffer (3 mM Tris pH 8.0; 0.15 mM EDTA pH 8.0; 3 mg lysozyme; 200 Units mutanolysin) and incubated for 30 min at 37°C with gentle agitation. Total RNA was isolated using the Rneasy Miny Kit (Qiagen, Hilden, Germany) as described by the manufacturer. Purified total RNAs were treated for 2 h at 37°C with 10 Units RQ1 RNase-Free DNase (Promega), and further purified on RNeasy mini columns (Qiagen, Hilden, Germany).

Reverse transcription reactions were performed in 20 µl volume, containing 1 µg total RNA, 500 ng random hexamers, 1 mM dNTPs, 3 mM MgCl_2_ and used in an ImProm-II Reverse Transcription System first-strand synthesis reaction (Promega Corp., USA) in the presence of 20 Units RNasin Ribonuclease Inhibitor. The reverse transcription reactions were first annealed at 25°C for 5 min and then incubated at 42°C for 120 min. Serial dilutions of the synthesized cDNA samples were prepared prior to qRT-PCR, which was performed using an MxPro Mx3000P Thermalcycler (Stratagene, Agilent Technologies, USA) and product accumulation was quantified by incorporation of SYBR Green. Primers for qRT-PCR were designed using the Primer 3 Quantification analysis software (see Table S1). Standard curves for each GAS strain were constructed by amplifying the *gyrA* gene. All samples were assayed at least in duplicate using 1 µl diluted cDNA with Brilliant SYBR Green QPCR Master Mix (Stratagene, Agilent Technologies, USA) and 1 µM concentrations of each gene-specific oligonucleotide. The amplification conditions were 10 min of denaturation at 95°C, 40 cycles of amplification (95°C for 30 sec; 45°C for 30 sec; 72°C for 30 sec), 1 cycle melting curve (95°C for 30 sec; 45°C for 30 sec; 95°C for 30 sec). All RT-PCRs amplified a single product as determined by melting curve analysis.

## Supporting Information

Figure S1 Aggregation and biofilm formation capacity of strain 43_M3 (FCT-3) grown at different pH conditions. A) Confocal Laser Scanning Microscopy micrographs of strain 43_M3 (FCT-3) grown for 12 hours on glass coverslips using C medium at a starting pH of 6.4 or 7.5 (magnification 60 x). B) Time-course OD600 measurement of 43_M3 (FCT-3) grown in tubes under static conditions using non-buffered C medium at a starting pH of 6.4 or 7.5. C) Picture of the same cultures taken after 12 hours showing precipitation of the bacteria grown at starting pH of 6.4.
(0.99 MB TIF)

Figure S2 Expression of pili in FCT-4 and FCT-1 CovS mutant isolates grown at different pH conditions. Immunoblot analysis of cell surface extracts of the GAS strain 2728_M12 (FCT-4), its mouse passaged CovS inactive mutant, and of the 3348_M1 (FCT-2) CovS inactive mutant strain. Bacteria were grown in non-buffered C-medium at starting pH values of 7.5 or 6.4 up to OD600 of 0.4; nitrocellulose transferred extracts were incubated with specific mouse polyclonal sera raised against specific AP-1 pilin proteins and Spy0269 protein.
(0.27 MB TIF)

Table S1 (0.04 MB DOC)

## References

[1] Cunningham MW (2000). Pathogenesis of group A streptococcal infections.. Clin Microbiol Rev.

[2] Musser JM, Shelburne SA (2009). A decade of molecular pathogenomic analysis of group A Streptococcus.. J Clin Invest.

[3] Kreikemeyer B, McIver KS, Podbielski A (2003). Virulence factor regulation and regulatory networks in Streptococcus pyogenes and their impact on pathogen-host interactions.. Trends Microbiol.

[4] Bessen DE, Manoharan A, Luo F, Wertz JE, Robinson DA (2005). Evolution of transcription regulatory genes is linked to niche specialization in the bacterial pathogen Streptococcus pyogenes.. J Bacteriol.

[5] Froehlich BJ, Bates C, Scott JR (2009). Streptococcus pyogenes CovRS mediates growth in iron starvation and in the presence of the human cationic antimicrobial peptide LL-37.. J Bacteriol.

[6] Shelburne SA, Keith D, Horstmann N, Sumby P, Davenport MT (2008). A direct link between carbohydrate utilization and virulence in the major human pathogen group A Streptococcus.. Proc Natl Acad Sci U S A.

[7] Gryllos I, Grifantini R, Colaprico A, Jiang S, Deforce E (2007). Mg(2+) signalling defines the group A streptococcal CsrRS (CovRS) regulon.. Mol Microbiol.

[8] Neely MN, Pfeifer JD, Caparon M (2002). Streptococcus-zebrafish model of bacterial pathogenesis.. Infect Immun.

[9] Akiyama H, Morizane S, Yamasaki O, Oono T, Iwatsuki K (2003). Assessment of Streptococcus pyogenes microcolony formation in infected skin by confocal laser scanning microscopy.. J Dermatol Sci.

[10] Cho KH, Caparon MG (2005). Patterns of virulence gene expression differ between biofilm and tissue communities of Streptococcus pyogenes.. Mol Microbiol.

[11] Baldassarri L, Creti R, Recchia S, Imperi M, Facinelli B (2006). Therapeutic failures of antibiotics used to treat macrolide-susceptible Streptococcus pyogenes infections may be due to biofilm formation.. J Clin Microbiol.

[12] Hanski E, Jaffe J, Ozeri V (1996). Proteins F1 and F2 of Streptococcus pyogenes. Properties of fibronectin binding.. Adv Exp Med Biol.

[13] Kreikemeyer B, Oehmcke S, Nakata M, Hoffrogge R, Podbielski A (2004). Streptococcus pyogenes fibronectin-binding protein F2: expression profile, binding characteristics, and impact on eukaryotic cell interactions.. J Biol Chem.

[14] Manetti AG, Zingaretti C, Falugi F, Capo S, Bombaci M (2007). Streptococcus pyogenes pili promote pharyngeal cell adhesion and biofilm formation.. Mol Microbiol.

[15] Abbot EL, Smith WD, Siou GP, Chiriboga C, Smith RJ (2007). Pili mediate specific adhesion of Streptococcus pyogenes to human tonsil and skin.. Cell Microbiol.

[16] Bessen DE, Kalia A (2002). Genomic localization of a T serotype locus to a recombinatorial zone encoding extracellular matrix-binding proteins in Streptococcus pyogenes.. Infect Immun.

[17] Kratovac Z, Manoharan A, Luo F, Lizano S, Bessen DE (2007). Population genetics and linkage analysis of loci within the FCT region of Streptococcus pyogenes.. J Bacteriol.

[18] Falugi F, Zingaretti C, Pinto V, Mariani M, Amodeo L (2008). Sequence variation in group A Streptococcus pili and association of pilus backbone types with lancefield T serotypes.. J Infect Dis.

[19] Koller T, Manetti AG, Kreikemeyer B, Lembke C, Margarit I Typing of the pilus-protein-encoding FCT region and biofilm formation as novel parameters in epidemiological investigations of Streptococcus pyogenes isolates from various infection sites.. J Med Microbiol.

[20] Loughman JA, Caparon M (2006). Regulation of SpeB in Streptococcus pyogenes by pH and NaCl: a model for in vivo gene expression.. J Bacteriol.

[21] Telford JL, Barocchi MA, Margarit I, Rappuoli R, Grandi G (2006). Pili in gram-positive pathogens.. Nat Rev Microbiol.

[22] Podbielski A, Woischnik M, Leonard BA, Schmidt KH (1999). Characterization of nra, a global negative regulator gene in group A streptococci.. Mol Microbiol.

[23] Nakata M, Podbielski A, Kreikemeyer B (2005). MsmR, a specific positive regulator of the Streptococcus pyogenes FCT pathogenicity region and cytolysin-mediated translocation system genes.. Mol Microbiol.

[24] Luo F, Lizano S, Bessen DE (2008). Heterogeneity in the polarity of Nra regulatory effects on streptococcal pilus gene transcription and virulence.. Infect Immun.

[25] Buccato S, Maione D, Rinaudo CD, Volpini G, Taddei AR (2006). Use of Lactococcus lactis expressing pili from group B Streptococcus as a broad-coverage vaccine against streptococcal disease.. J Infect Dis.

[26] Edwards AM, Manetti AG, Falugi F, Zingaretti C, Capo S (2008). Scavenger receptor gp340 aggregates group A streptococci by binding pili.. Mol Microbiol.

[27] Fogg GC, Gibson CM, Caparon MG (1994). The identification of rofA, a positive-acting regulatory component of prtF expression: use of an m gamma delta-based shuttle mutagenesis strategy in Streptococcus pyogenes.. Mol Microbiol.

[28] Mora M, Bensi G, Capo S, Falugi F, Zingaretti C (2005). Group A Streptococcus produce pilus-like structures containing protective antigens and Lancefield T antigens.. Proc Natl Acad Sci U S A.

[29] Lizano S, Luo F, Bessen DE (2007). Role of streptococcal T antigens in superficial skin infection.. J Bacteriol.

[30] Courtney HS, Ofek I, Penfound T, Nizet V, Pence MA (2009). Relationship between expression of the family of M proteins and lipoteichoic acid to hydrophobicity and biofilm formation in Streptococcus pyogenes.. PLoS One.

[31] Dong YM, Pearce EI, Yue L, Larsen MJ, Gao XJ (1999). Plaque pH and associated parameters in relation to caries.. Caries Res.

[32] Moscoso M, Garcia E, Lopez R (2006). Biofilm formation by Streptococcus pneumoniae: role of choline, extracellular DNA, and capsular polysaccharide in microbial accretion.. J Bacteriol.

[33] Ehlers C, Ivens UI, Moller ML, Senderovitz T, Serup J (2001). Females have lower skin surface pH than men. A study on the surface of gender, forearm site variation, right/left difference and time of the day on the skin surface pH.. Skin Res Technol.

[34] Rippke F, Schreiner V, Schwanitz HJ (2002). The acidic milieu of the horny layer: new findings on the physiology and pathophysiology of skin pH.. Am J Clin Dermatol.

[35] Rippke F, Schreiner V, Doering T, Maibach HI (2004). Stratum corneum pH in atopic dermatitis: impact on skin barrier function and colonization with Staphylococcus Aureus.. Am J Clin Dermatol.

[36] Simmen HP, Giovanoli P, Battaglia H, Wust J, Meyer VE (1995). Soft tissue infections of the upper extremities with special consideration of abscesses in parenteral drug abusers. A prospective study.. J Hand Surg Br.

[37] Marsh PD (2006). Dental plaque as a biofilm and a microbial community - implications for health and disease.. BMC Oral Health.

[38] Donlan RM, Costerton JW (2002). Biofilms: survival mechanisms of clinically relevant microorganisms.. Clin Microbiol Rev.

[39] Beenken KE, Dunman PM, McAleese F, Macapagal D, Murphy E (2004). Global gene expression in Staphylococcus aureus biofilms.. J Bacteriol.

[40] Regassa LB, Novick RP, Betley MJ (1992). Glucose and nonmaintained pH decrease expression of the accessory gene regulator (agr) in Staphylococcus aureus.. Infect Immun.

[41] Weinrick B, Dunman PM, McAleese F, Murphy E, Projan SJ (2004). Effect of mild acid on gene expression in Staphylococcus aureus.. J Bacteriol.

[42] Rice KC, Nelson JB, Patton TG, Yang SJ, Bayles KW (2005). Acetic acid induces expression of the Staphylococcus aureus cidABC and lrgAB murein hydrolase regulator operons.. J Bacteriol.

[43] O'Neill E, Pozzi C, Houston P, Humphreys H, Robinson DA (2008). A novel Staphylococcus aureus biofilm phenotype mediated by the fibronectin-binding proteins, FnBPA and FnBPB.. J Bacteriol.

[44] Hamilton IR, Svensater G (1998). Acid-regulated proteins induced by Streptococcus mutans and other oral bacteria during acid shock.. Oral Microbiol Immunol.

[45] Santi I, Grifantini R, Jiang SM, Brettoni C, Grandi G (2009). CsrRS regulates group B Streptococcus virulence gene expression in response to environmental pH: a new perspective on vaccine development.. J Bacteriol.

[46] Fogg GC, Caparon MG (1997). Constitutive expression of fibronectin binding in Streptococcus pyogenes as a result of anaerobic activation of rofA.. J Bacteriol.

[47] Granok AB, Parsonage D, Ross RP, Caparon MG (2000). The RofA binding site in Streptococcus pyogenes is utilized in multiple transcriptional pathways.. J Bacteriol.

[48] Nakata M, Koller T, Moritz K, Ribardo D, Jonas L (2009). Mode of expression and functional characterization of FCT-3 pilus region-encoded proteins in Streptococcus pyogenes serotype M49.. Infect Immun.

[49] Kreikemeyer B, Nakata M, Koller T, Hildisch H, Kourakos V (2007). The Streptococcus pyogenes serotype M49 Nra-Ralp3 transcriptional regulatory network and its control of virulence factor expression from the novel eno ralp3 epf sagA pathogenicity region.. Infect Immun.

[50] Beckert S, Kreikemeyer B, Podbielski A (2001). Group A streptococcal rofA gene is involved in the control of several virulence genes and eukaryotic cell attachment and internalization.. Infect Immun.

[51] Li L, Jia Y, Hou Q, Charles TC, Nester EW (2002). A global pH sensor: Agrobacterium sensor protein ChvG regulates acid-inducible genes on its two chromosomes and Ti plasmid.. Proc Natl Acad Sci U S A.

[52] Pflock M, Kennard S, Finsterer N, Beier D (2006). Acid-responsive gene regulation in the human pathogen Helicobacter pylori.. J Biotechnol.

[53] Perez JC, Groisman EA (2007). Acid pH activation of the PmrA/PmrB two-component regulatory system of Salmonella enterica.. Mol Microbiol.

[54] Rosch JW, Mann B, Thornton J, Sublett J, Tuomanen E (2008). Convergence of regulatory networks on the pilus locus of Streptococcus pneumoniae.. Infect Immun.

[55] Dalton TL, Scott JR (2004). CovS inactivates CovR and is required for growth under conditions of general stress in Streptococcus pyogenes.. J Bacteriol.

[56] Lyon WR, Gibson CM, Caparon MG (1998). A role for trigger factor and an rgg-like regulator in the transcription, secretion and processing of the cysteine proteinase of Streptococcus pyogenes.. EMBO J.

