# Supplementary figures and images for: Environmental Acidification Drives S. pyogenes Pilus Expression and Microcolony Formation on Epithelial Cells in a FCT-Dependent Manner
Source: PLoS One. 2010 Nov 5;5(11):e13864. doi: 10.1371/journal.pone.0013864 (PMC2974651; doi:10.1371/journal.pone.0013864)

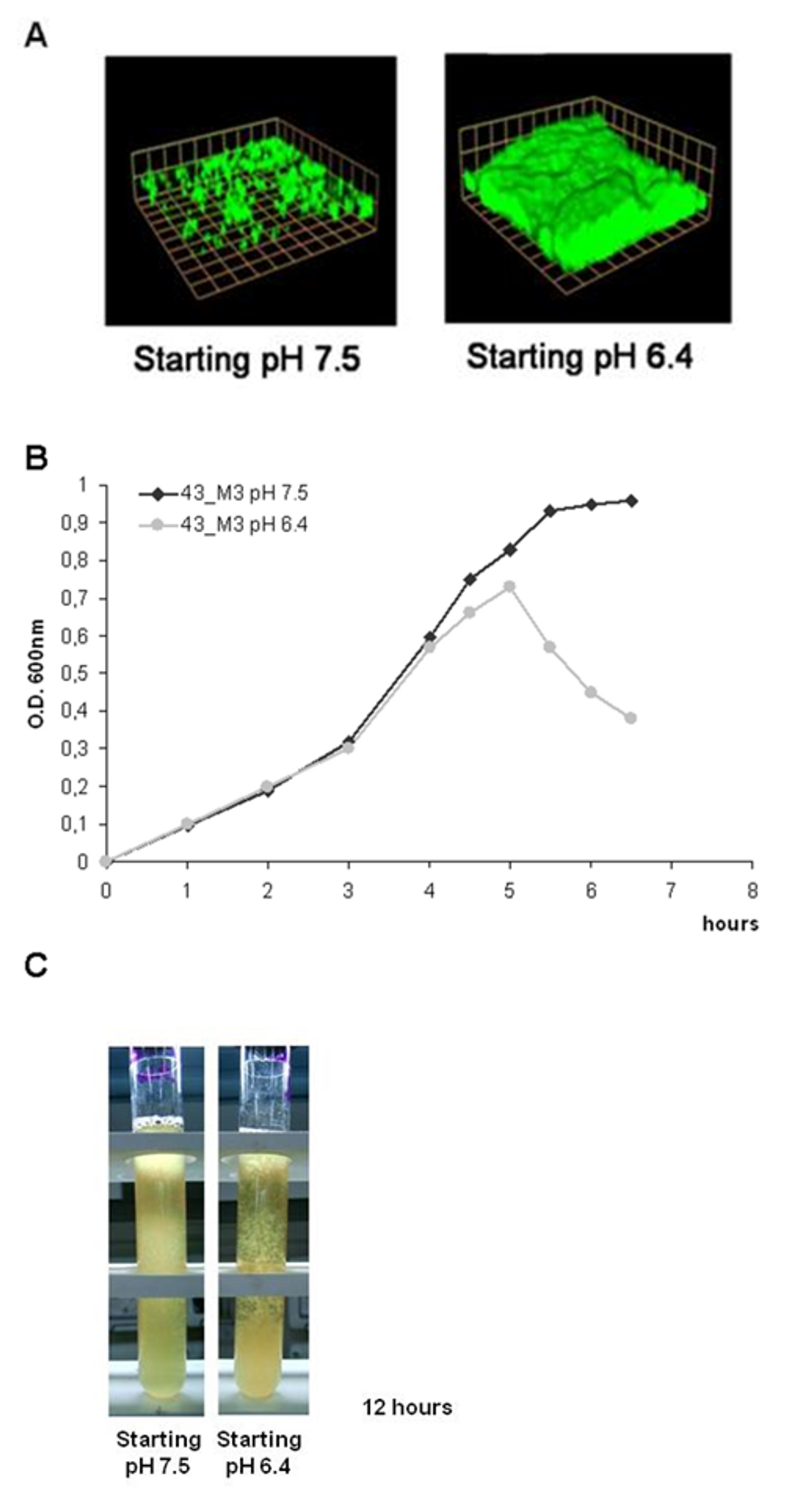

Supplement: Figure S1 — Aggregation and biofilm formation capacity of strain 43_M3 (FCT-3) grown at different pH conditions. A) Confocal Laser Scanning Microscopy micrographs of strain 43_M3 (FCT-3) grown for 12 hours on glass coverslips using C medium at a starting pH of 6.4 or 7.5 (magnification 60 x). B) Time-course OD600 measurement of 43_M3 (FCT-3) grown in tubes under static conditions using non-buffered C medium at a starting pH of 6.4 or 7.5. C) Picture of the same cultures taken after 12 hours showing precipitation of the bacteria grown at starting pH of 6.4. (0.99 MB TIF) [file pone.0013864.s001.tif]

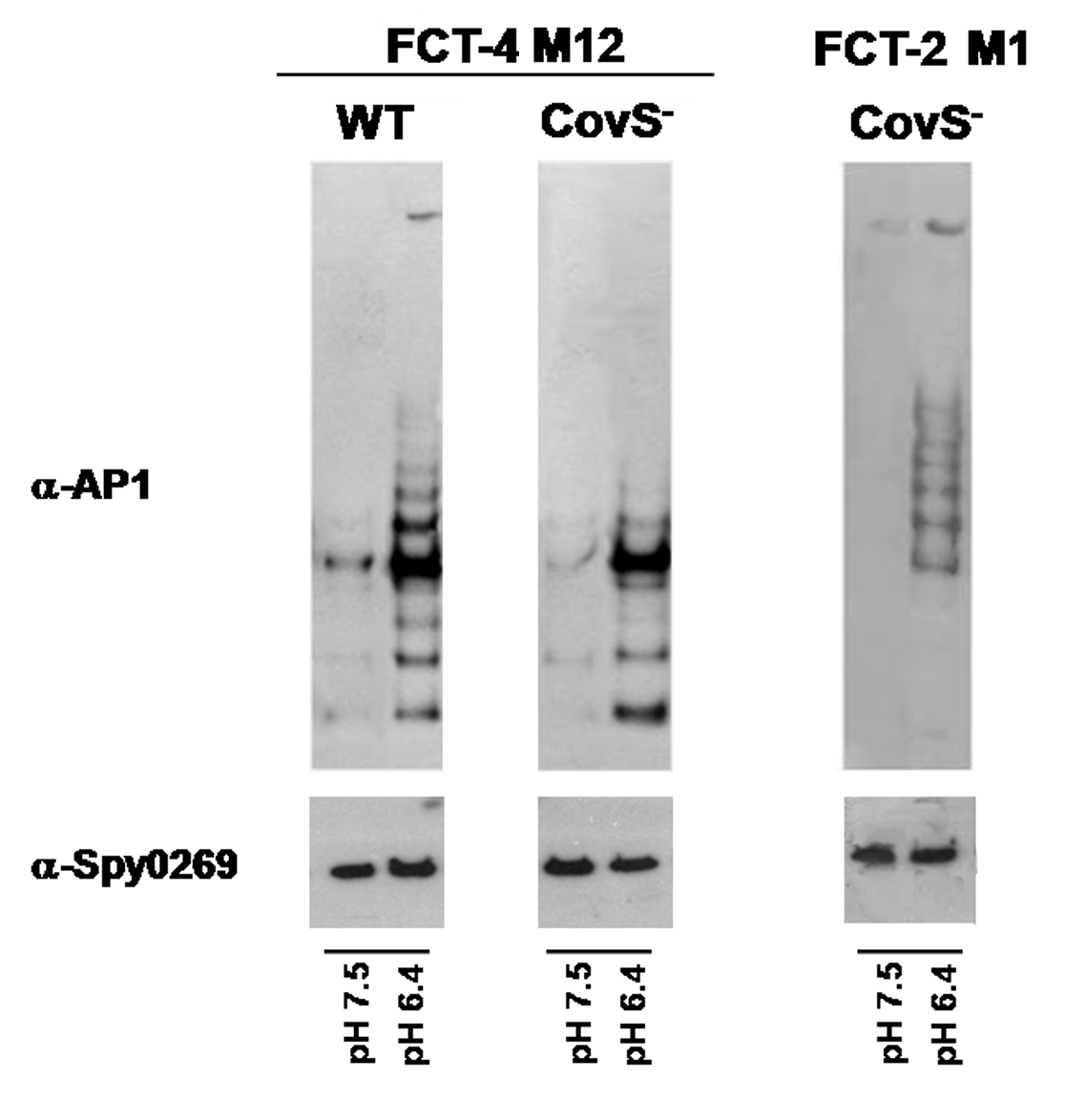

Supplement: Figure S2 — Expression of pili in FCT-4 and FCT-1 CovS mutant isolates grown at different pH conditions. Immunoblot analysis of cell surface extracts of the GAS strain 2728_M12 (FCT-4), its mouse passaged CovS inactive mutant, and of the 3348_M1 (FCT-2) CovS inactive mutant strain. Bacteria were grown in non-buffered C-medium at starting pH values of 7.5 or 6.4 up to OD600 of 0.4; nitrocellulose transferred extracts were incubated with specific mouse polyclonal sera raised against specific AP-1 pilin proteins and Spy0269 protein. (0.27 MB TIF) [file pone.0013864.s002.tif]
