# Supplementary material for: Environmental Acidification Drives S. pyogenes Pilus Expression and Microcolony Formation on Epithelial Cells in a FCT-Dependent Manner
Source: PLoS One. 2010 Nov 5;5(11):e13864. doi: 10.1371/journal.pone.0013864 (PMC2974651; doi:10.1371/journal.pone.0013864)
Supplement: Table S1 — (0.04 MB DOC) [file pone.0013864.s003.doc]

Supplementary Table 1. Oligonucleotide primers used for RT-PCR experiments

| **Primer name** | **Primer sequence** | **Tm (°C)** | **PCR product lenght (bp)** |
| --- | --- | --- | --- |
| CPAM1 F | AAGGCTTGGAACCCTTGAAT | 54 | 209 |
| CPAM1 R | CGGAACTTGTTTTGGCATTTC | 54 |
| ROFM1 F | GTTTTTGTCTTGCTGCGTCA | 54 | 215 |
| ROFM1 R | CTCGTCCCCTTACGGATTTT | 54 |
| CPAM6 F | CAATGGTACCAATTATCATGCTG | 52 | 208 |
| CPAM6 R | TATTGCAAGTTTGCTTCCTTCTTG | 52 |
| F1M6 F | ATCCCCCTAAACCTGGTGAG | 54 | 163 |
| F1M6 R | CTCTCGCTTGGAACTTCTGG | 54 |
| ROFM6 F | CGGCATCAATTTTCGGTAAC | 53 | 199 |
| ROFM6 R | TCCATTGTAAGCTCGCAAAA | 53 |
| CPAM3 F | AGGCTCCTATTATTCCTATTACTC | 52 | 204 |
| CPAM3 R | GATTCTCCATCCTTTAATGTGAAG | 52 |
| F2 F | CTCACGCCCAGTTGATACCT | 54 | 248 |
| F2 R | CTGCGGTTTCGACAAATGTA | 50 |
| MSMRL F | GTGCGGAACCTTTTGATAGC | 52 | 235 |
| MSMRL R | CTTGCTTGAGCAATTGTGGA | 50 |
| NRAM3 F | GTTTTTGTCTTGCTGCGTCA | 54 | 215 |
| NRAM3 R | CTCGTCCCCTTACGGATTTT | 54 |
| GYRA_F | GTCTTGCTCAAGATGAATTT | 52 | 242 |
| GYRA_R | TAGAAGATGGTGAAACCATT | 52 |
